# Navigating chronic uncertainty: a theory synthesis for nursing communication in life-limiting illness

**DOI:** 10.1186/s12912-024-02328-7

**Published:** 2024-09-13

**Authors:** Julie B. Grant, Rachel Johnson-Koenke

**Affiliations:** College of Nursing, University of Colorado Anschutz Medical Campus, Aurora, 80045 USA

**Keywords:** Communication, Nursing, Palliative care, Theory synthesis, Uncertainty

## Abstract

**Background:**

Despite the proven benefits of early palliative care, patient communication regarding these services remains elusive. Therefore, this paper aims to (a) provide a focused literature review on nurse palliative care communication addressing chronic uncertainty in life-limiting illness (LLI), (b) define the Reconceptualization of Uncertainty in illness Theory and Problematic Integration Theory within a nursing Unitary Caring Science philosophical worldview and, (c) synthesize these theories and literature review into a unique theoretical framework for early palliative care communication in acute care nursing.

**Method:**

Turner’s theory synthesis methodology was combined with a PRISMA-style literature review. The literature search was conducted in July 2023 and updated in May 2024 using EBSCOhost, Pubmed, and PsychINFO databases. A second literature search was undertaken to identify applicable uncertainty theories in chronic or LLIs.

**Results:**

This theory synthesis highlights the interconnectedness of all facets of uncertainty for those living with severe LLI (personal factors, types of uncertainty, and the nursing communicatory process) and conceptualizes uncertainty communication as a series of events happening simultaneously, not simply a cause-and-effect process.

**Discussion:**

The framework resulting from this synthesis encourages nurses’ holistic understanding of the complex nature of uncertainty in LLI, highlights the integral communicatory role nurses have in their patients’ health and wellness, and promotes further nursing-specific communication research. Future research on enabling nurse-initiated early palliative care communication and narrative communication techniques will support patients’ values and dignity throughout more than a single hospital stay but their entire disease trajectory.

**Supplementary Information:**

The online version contains supplementary material available at 10.1186/s12912-024-02328-7.

Many theories within health communication offer valuable insights for nursing and early palliative care communication. However, while various research disciplines study communication and have subsequently developed theories, nursing must recognize and research its own unique communicatory role. Therefore, this paper aims to (a) provide a focused literature review on nurse palliative care communication addressing chronic uncertainty in life-limiting illness (LLI), (b) define the Reconceptualization of Uncertainty in illness Theory (RUIT) and Problematic Integration Theory (PIT) within a nursing Unitary Caring Science (UCS) worldview and, (c) synthesize these theories and literature review into a unique theoretical framework for early palliative care communication in acute care nursing (ACN).

## Background

### Early palliative care

Early palliative care prevents and relieves suffering by identifying and treating pain and other physical, psychosocial, and spiritual problems in patients with serious LLI [1, 2]. Research demonstrates that early palliative care increases quality-of-life and goals-of-care discussions while decreasing emergency room visits, improving satisfaction with care, and reducing healthcare costs [3–5]. Additionally, Temel et al.’s (2010) landmark study on early palliative care found that patients with metastatic non-small-cell lung cancer who received early palliative care had longer median survival rates than patients who received more aggressive cancer treatments [5]. Despite the overwhelming benefits of early palliative care and national initiatives to increase access, widespread adoption of these services remains elusive [6, 7]. Palliative care is not occurring until late in patients’ disease trajectories, when they transition away from curative treatment, experience uncontrolled symptoms, or concurrently with hospice referrals [6]. Furthermore, existing research highlights ACNs’ lack of understanding regarding the current definition of palliative care, uncertainty in communicating with their patients about quality of life, and ambiguity surrounding their scope of practice [8–10].

### Unitary caring science

Jean Watson’s UCS philosophy aligns with a unitary transformative worldview that highlights the interconnectedness of all aspects of existence, including people, their environments, and the ever-evolving universe [11–13]. Watson’s philosophy of caring emphasizes that human caring should be regarded as an end in itself rather than merely a means to a medical cure [11]. UCS’s focus on a moral-ethical perspective – emphasizing human dignity, wholeness, caring, and connectedness – makes it a widely embraced theoretical framework in nursing palliative care research [11, 14–16]. Additionally, UCS’s concepts of *authentic presence* and *transpersonal caring moments* offer valuable insights into early palliative care communication. Authentic presence involves a nurse’s complete and genuine engagement with the patient’s entire being, encompassing subjective, objective, reflective, aesthetic, ethical, and spiritual dimensions of knowing [11, 15]. This genuine, caring presence fosters a deeper understanding of the patient’s wellbeing throughout their health and chronic illness journey.

### Research gaps

Currently, the effects of ACN communication on subsequent palliative care discussions focus primarily on nurses’ perceived barriers to communication as opposed to their strengths [8, 9, 17, 18]. ACNs’ are ideally situated to initiate early palliative care discussions because they frequently care for patients with serious LLI *before* critical care is necessary [19]. Yet, most palliative care communication research focuses on palliative care teams and specialty nurses [20, 21]. Physician perspectives drove previous approaches to communication training and mainly addressed the stress of imparting bad news [22]. However, the ACN’s role is fundamentally different, involving communication once uncertainty has been introduced and providing care as uncertainty ebbs and flows throughout a lifelong illness trajectory [20]. Despite substantial ACN-patient interaction and unique insight into their patients’ holistic wellbeing, little palliative care communication research focuses on this population [10, 23, 24]. Subsequently, the following theory synthesis of RUIT, PIT, UCS, and current research on nursing palliative communication addressing chronic uncertainty supports a theoretical framework to guide nurses in understanding this complex phenomenon.

The authors of this theory synthesis are from the United States and have nursing, social work, and palliative care backgrounds. Consequently, the findings are inherently interpreted through the authors’ professional and cultural perspectives. The following theory synthesis aims to (a) provide a focused literature review on nurse palliative care communication addressing chronic uncertainty in LLI, (b) define the RUIT and PIT within a nursing UCS worldview, and (c) synthesize these theories and literature review into a unique theoretical framework for early palliative care communication in ACN.

## Method

Turner’s theory synthesis methodology was combined with a Preferred Reporting Items for Systematic Reviews and Meta-analysis (PRISMA) style literature review [25]. We condensed Turner’s steps for theory synthesis and included a focused literature review on palliative care communication addressing chronic uncertainty in LLI to provide additional context and produce an “overarching framework that transcends and interprets existing theories” [26]. (Table 1). The literature review supplemented and informed our theory synthesis and did not generate new theory in and of itself. Watson’s UCS philosophy further guided this research, with literature and uncertainty theories interpreted through a caring science nursing lens.

**Table 1:** Methodological steps for theory synthesis

Step	Description
Step 1: Literature review	Focused review of existing literature Review of applicable theories
Step 2: Synthesis preparation	Clarify existing theories Extract what is useful, plausible, and relevant
Step 3: Synthesis	Break down theories into simplest propositions Compare theories for convergence and divergence Bring together aspects of convergence
Step 4: Synthesis refinement	Examine relationships and causal processes Maintain view of generating further theoretical insights

### Search strategy and selection criteria

We utilized Covidence software to track and organize our literature review and PRISMA formatting to structure our results [25]. A focused literature search was conducted in July of 2023 and updated in May 2024 using EBSCOhost, PsychINFO, and Pubmed databases. We used variations of the following Boolean phrase: (((“Communication“[Mesh] OR “Social Communication*” OR Misinformation OR “Personal Communication” OR “Communication Program*” OR “Communication Program” OR “Communications Personnel” OR “Communication Barrier*“[Mesh]) AND (“Nurs*“[Mesh])) AND (Uncertainty)) AND (“Palliative Care“[Mesh] OR “Palliative Treatment*” OR “life-limiting” OR “chronic illness” OR “chronic disease” OR “Palliative Therap*” OR “Palliative Supportive Care” OR “Palliative Surger*”). The asterisk in this Boolean phrase is a truncation operator that includes alternative endings for those words. Inclusion for the literature review comprised publications addressing palliative care, patient or primary caregiver uncertainty, and applicability to ACN communication. Exclusion criteria included ongoing research, full text not available in English, failure to address how communication relates to patient or caregiver uncertainty, articles greater than 20 years old, and ongoing research (Table 2). Search results were limited to the last 20 years because the American Board of Medical Specialties only recognized palliative care as a specialty in 2006, with the patient population considered appropriate for palliative care increasing dramatically since then [27]. A second reviewer verified the applicability of the selected articles. The article selection process used no automation tools or artificial intelligence functionalities.

**Table 2:** Inclusion and exclusion criteria

Inclusion	Exclusion
Addresses patients who have LLI or severe chronic illness	Does not address how communication relates to patient or caregiver uncertainty
Addresses patient or primary caregiver uncertainty	Full text unavailable in English language
Addresses Registered Nurse communication	Ongoing research
	Greater than 20 years old

A second literature search was undertaken to identify applicable uncertainty theories in chronic or LLI. This theory-oriented search used health communication and nursing theory textbooks, reference chaining from our first comprehensive literature review, and keyword searches of applicable theories in EBSCOhost, PsychINFO, and Pubmed databases. While many nursing and health communication theories addressed aspects of uncertainty in illness, such as harm reduction, uncertainty reduction, uncertainty management, and motivated information management, only the RUIT and PIT explicitly addressed aspects of uncertainty in chronic or LLI [28–31].

### Data analysis

**Fig. 1:**
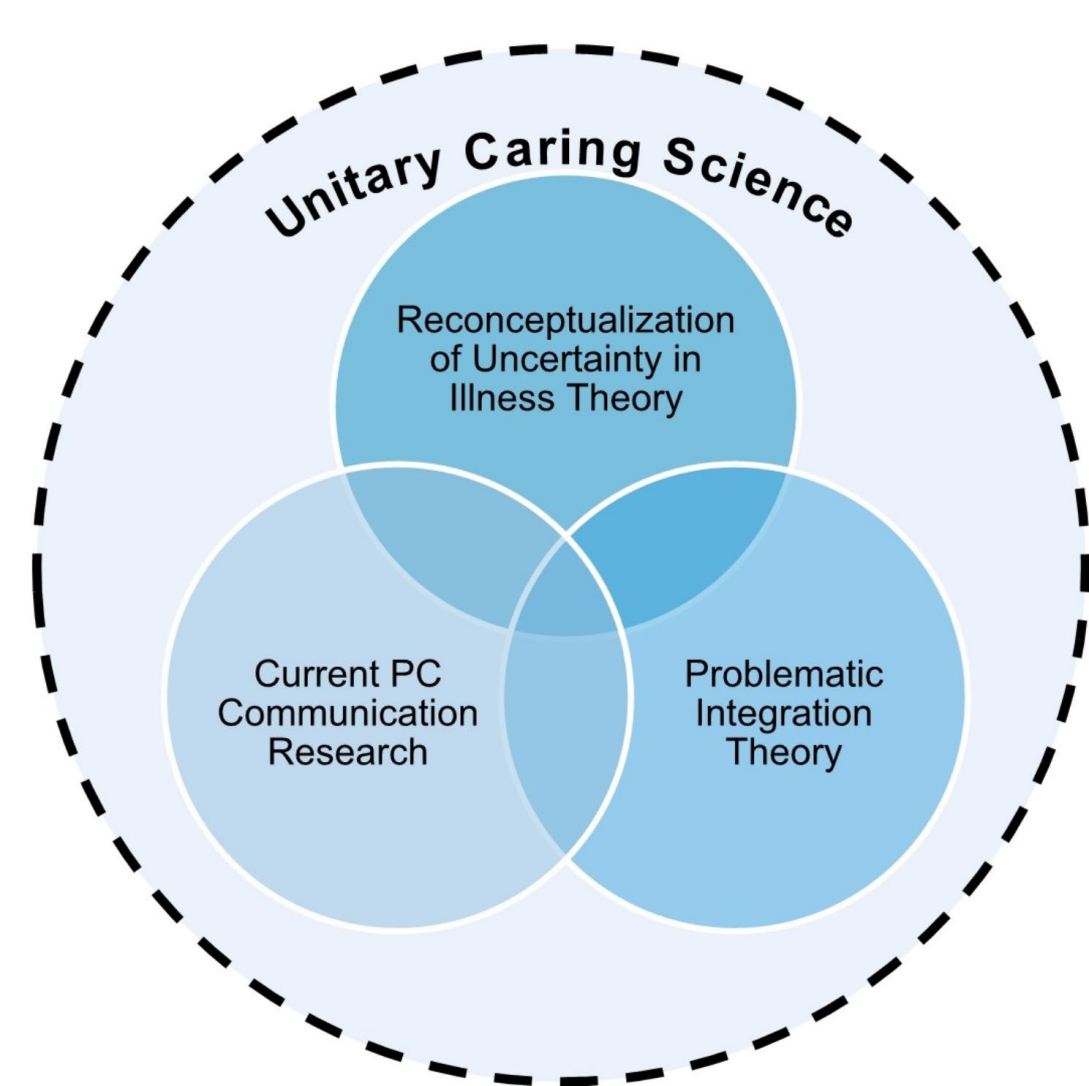
Visualization of theory synthesis

Data analysis integrated our literature review with our analysis of RUIT, PIT, and UCS (Fig. 1). We initiated our data analysis through an iterative reading and rereading of all included uncertainty in chronic illness literature. For the research article literature review, we utilized an inductive approach using content analysis techniques to stay close to the data while deriving significant themes, concepts, and ideas that would apply to our subsequent theory synthesis [32, 33]. Our theoretical synthesis method closely followed Turner’s steps (Table 1). We organized the theories into a tabular form to facilitate a crosswalk analysis comparing the convergence and divergence of concepts regarding the overarching topic of ACN early palliative care communication and chronic uncertainty in LLI.

## Results

### Step one: literature review search results

**Fig. 2:**
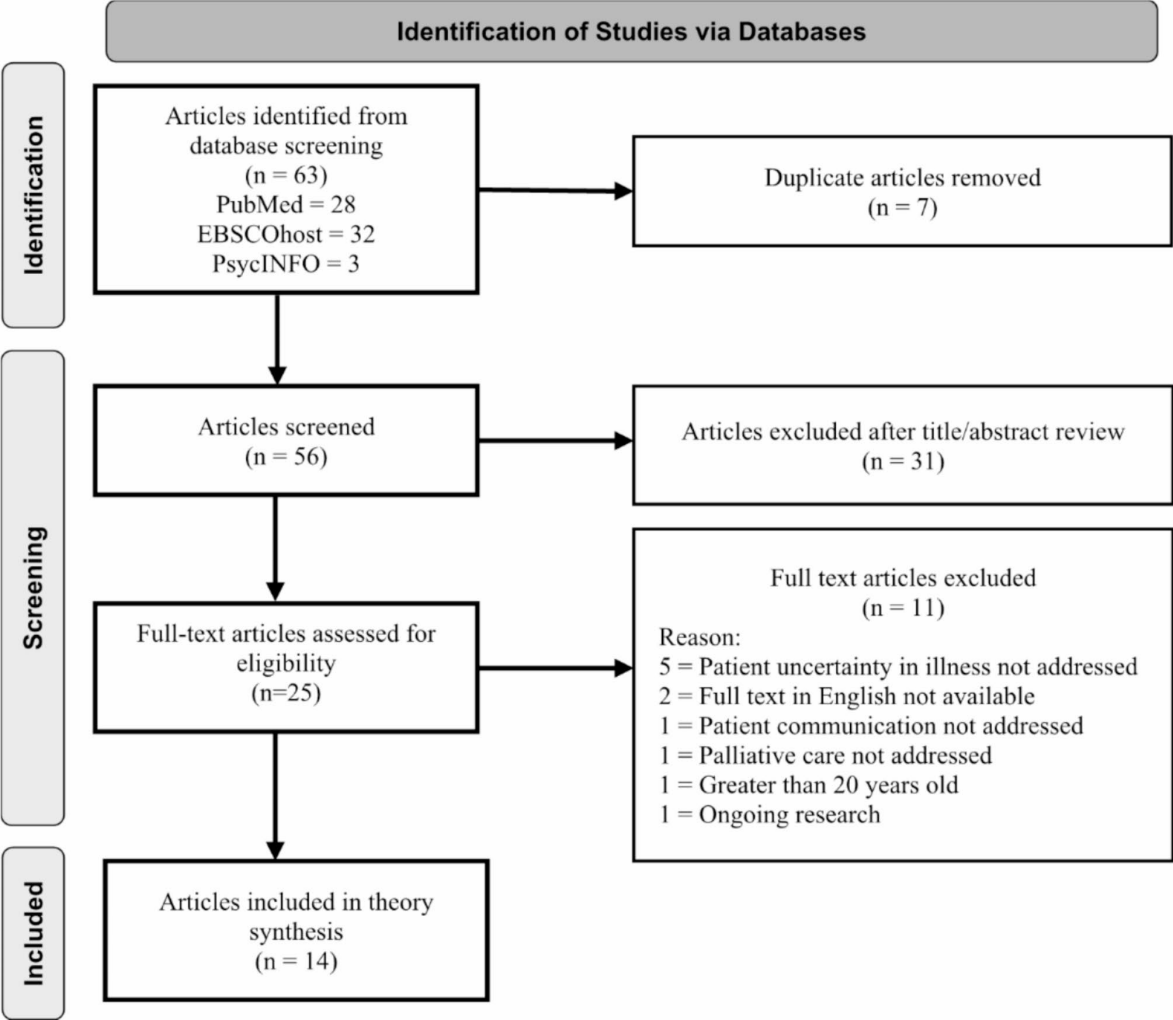
PRISMA diagram of the selection of studies on uncertainty palliative care communication

While a formal literature review is not an explicit step in Turner’s methodology, we initiated our synthesis with this step to better understand existing uncertainty research as it directly relates to nursing, locate uncertainty theories, and compare the plausibility of prospective uncertainty theories to current research [26]. Database searches yielded 63 articles: 28 publications from PubMed, 32 from EBSCOhost, and three from PsycINFO. After removing seven duplicate articles and reviewing the initial title and abstract, 25 articles remained eligible for full-text review. During the full-text review, 11 publications lacked inclusion criteria. Subsequently, 14 articles remained applicable for inclusion in this theory synthesis (Fig. 2). Next, we compiled a matrix of the relevant literature to highlight the article’s contributions to ACN communication and uncertainty in LLI (Table 3).

**Table 3:** Nursing communication in life-limiting illness literature matrix and synthesis

Author/Year	Purpose	Country	Method	Participants	Summary of Findings	Theory Synthesis Contribution
Al-Yateem et al., 2017 [34]	Assess uncertainty felt by parents of children with chronic conditions	United Arab Emirates	Exploratory descriptive, cross-sectional design	*N* = 204 parents with child	- Most participants had moderate to high uncertainty and 5% reported low uncertainty - Factors contributing to higher uncertainty were: did not speak local language, child < 1 year old, child acutely ill, child approaching puberty	- Biopsychosocial ecological model represented in the attributes of higher uncertainty - Social determinants of health = different areas of the emirates had widely differing levels of uncertainty - Uncertainty inherent to chronic conditions
Bélanger et al., 2016 [35]	Understand how patient participation in palliative care decisions is constructed through discourse	Canada	Discourse analysis Social constructionist epistemological position	*N* = 26 18 palliative care patients 6 family physicians 2 nurses	- Interpretive repertoires to justify patient interaction include: Exposing uncertainty, co-constructing patient preferences, affirming patient autonomy, and upholding authority of healthcare providers	- Rarely explicit decision-making interaction. - Interpretive repertoires used to covertly negotiate roles in decision-making
Burke et al., 2023 [36]	Explore perspectives of healthcare practitioners providing palliative care to patients from culturally diverse backgrounds	Ireland	Qualitative systematic review	*N* = 26 articles	- Practitioners emphasized communication and connection, the important role of the family in death and dying, challenges in their role of education that addresses uncertainty, and that institutional and societal factors impact their ability to communicate	- Fear of acting in a culturally insensitive way - Family role and individualized care is crucial - Need for communication and palliative care education. - Provider uncertainty impacted patient uncertainty
Checton et al., 2012 [37]	Examine couples experiences of uncertainty related to one partner’s chronic illness and develop a model of communication efficacy	United States	Survey	*N* = 616 people, or 308 couples in a committed relationship where one partner has a chronic health condition	- Prognosis and symptom uncertainty influence how couples managed chronic health conditions - Unpredictable symptom burden was negatively associated with communication - Couples who shared information about the chronic illness were more likely to report they were managing the condition well	- The more uncertain the partner was about a prognosis, the more they perceived the illness’s interference in their lives – illness interference then negatively impacted communication - Symptom uncertainty was a significant negative predictor of communication efficacy for patients, not partners
Formagini et al., 2022 [38]	Explore what obscures or clarifies the stigma around palliative care and its associations with end of life in cancer care	United States	Qualitative grounded theory	*N* = 44 18 patients 13 caregivers 13 non-PC oncology professionals	- Patients and caregivers apprehensive about palliative care referrals because of their association with end-of-life and were more confident about referrals when confident about their prognosis, familiar with palliative care already, and oncology team used clear communication around palliative care referral - Social network perceptions of palliative care impacted patient acceptance.	- Proactive communication about palliative care decreases uncertainty - Clear communication around prognosis may not decrease uncertainty but do increase acceptance of palliative care options
Gofton et al., 2018 [39]	Develop an understanding of health practitioners’ conceptualization of palliative care in neurology	Canada	Qualitative thematic analysis Grounded Theory	*N* = 4 Focus groups (physicians, nursing, allied health, trainees) Semi-structured interviews (patient-caregiver dyads)	- Practitioners face communication barriers arising from neuro disease - Practitioners face challenges due to: uncertainty with prognosis, lack of support availability, variable disease trajectory, inconsistency in information between providers, and attitudes and skills of caregivers - Providers perceive patients’ existential distress (emotional, psychological, spiritual) results from function loss and a threat to personhood from neuro symptom burden	- The communication skills of the provider and availability of support systems for the patient influence uncertainty - Communication itself, is impacted by the disease and impacts uncertainty
Harrison et al., 2021 [40]	Describe palliative care needs and experiences of people with mesothelioma and their caregivers	United Kingdom	Integrative systematic review	*N* = 14 articles	- Uncertainty – theme cut across all other categories - People with mesothelioma have many communication needs including organization of services, information, management of care, caregiver, and legal, which all impact feelings of uncertainty - People want a “named point of contact” - Carers valued the benefits of early palliative care more than patients	- Uncertainty took away feelings of control - Legal issues associated with mesothelioma compensation uniquely contributed to this population’s uncertainty - Caregiver uncertainty was distinct from patient uncertainty
Hendricks-Ferguson et al., 2021 [41]	Describe the development and testing of a palliative care/EOL communication intervention (COMPLETE)	United States	Pilot study followed by randomized control trial	*N* = 13 parents of 11 children with high-grade malignant brain tumors.	- MD/RN dyad used conversation guide and visual aids to help parents identify child’s values and make realistic goals-of-care in context of shifting prognosis. - Intervention feasible and acceptable to parents - After session, parents reported increased hope, decrease uncertainty, diminished pain and procedural anxiety based on parent perceptions, and more meaningful dialogue with oncology providers and discussions regarding EOL before health crisis	- Improved communication techniques in one meeting allowed parents to communicate better with other providers
Kelley et al., 2023 [42]	Explore uncertainty of patients and providers in the older ICU trauma patient population	United States	Prospective observational (survey)	*N* = 100 trauma patients 50 years and older admitted to the ICU 39 patients 61 patient surrogates	- Significant increase in uncertainty if surrogate answered questions or if no prior discussions about EOL - Nurse, resident, and attending predictions on 6-month survival similar but thoughts on initiating palliative care consult differed (nurses said yes 27%, physicians only said yes 18%)	- Early communication decreased uncertainty by addressing end of life when patients could still participate - Nurses are more likely to support palliative care earlier
King et al., 2004 [43]	Explore caregivers’ experiences of out-of-hours care and services	United Kingdom	Qualitative Semi-structured interviews Thematic analysis	*N* = 15 bereaved carers	- Caregivers felt well-supported out-of-hours, especially by nursing services, and liked when they could form relationships with staff who worked out-of-hours - Problems were associated with: poor provision of information, inadequate communication, difficulty accusing night services, or inflexibility of services - When everyone on the healthcare team was not updated on the patient’s status, it caused caregiver distress – having to continually recount a problem/concern	- Knowing who to contact out-of-hours provided comfort in uncertainty - Nurses were the primary contact for caregivers after hours, and their communication often impacted carers’ immediate concerns - Anticipatory approach to communication and care decreased uncertainty - Negative experiences increased uncertainty and related back to poor communication and inflexible service provision
Koffman et al., 2019 [44]	Optimize and determine the feasibility of a cluster randomized controlled trial of AMBER care	United Kingdom	Randomized controlled trial.	*N* = 65 participants 36 = intervention 29 = standard care 24 interviews (patients) 4 focus groups (providers) 15 observations (team meetings) 6 case note reviews 3 heat maps 15 bereavement surveys completed	- Emphasis on “clinical uncertainty” prompted healthcare professional awareness of often-overlooked patients. - The AMBER care bundle among acutely unwell patient populations was possible but not practical or feasible. Needs simplification and optimization	- Instead of looking solely for disease progression or severity as an indicator for early palliative care, providers looked for uncertainty in the patient’s clinical trajectory as an indicator for palliative care - The more complicated, the more uncertain
Larkin et al., 2007 [45]	Explore the patient perspective of palliative care transition for advanced cancer patients in Europe	Belgium	Qualitative Phenomenological approach	*N* = 100 advanced cancer patients in 6 European countries 20 = UK 20 = IRL 15 = NL 18 = I 10 = SP 17 = CH	- With transitions, there were: mixed messages, poor communication, and increased uncertainty - Physical environment of Hospice offered ontological security from which to address concerns with transitions. - Patients oscillated between reality of their now and an uncertain future - Integrating palliative care into acute care hospital units makes it less visible and less palliative-the influence of acute care overpowers the palliative care - “Transience” – the action or fact of passing away, offered researchers a clearer description of the transition experience (limited durability and impermanence)	- Transience accounts for the inevitable uncertainty of life-limiting illness. Uncertainty is always there, yet fluctuating with location, time, and circumstances
O’Connell et al., 2003 [46]	Determine caregiver perspectives on support and educational needs for stroke survivors in acute and community settings	Australia	Qualitative Exploratory descriptive Semi structures interviews	*N* = 28 caregivers 14 from acute setting 14 from community setting	- High levels of uncertainty in acute and community settings for caregivers after stroke but *especially* acute care, due to an abrupt placement in an unfamiliar role and uncertainty in what was immediately happening to their family member and what to expect next - Limited information with poor continuity of care increased uncertainty. - Caregivers indicated they were required to provide patients with financial support, emotional support, company, and physical support, which many were not equipped or given the resources to provide	- Caregivers had to actively seek information to reduce their uncertainty; information was not routinely given - Difficulty accessing health professionals greatly impacted uncertainty
Schulman-Green et al., 2023 [47]	Test *Managing Cancer Care: A Personal Guide* to improve palliative care literacy and cancer self-management	United States	Single-blind randomized controlled trial	*N* = 71 Stage I-IV breast cancer patients age 21 + with > 6 month prognosis. 32 = intervention 39 = standard care	- The intervention group largely improved their palliative care literacy. Late stage patients showed increased self-management and reduced anxiety, depression, and uncertainty - Intervention was feasible and effective in late stage cancer	- Educating and empowering patients for self-management can reduce uncertainty and improve emotional outcomes regardless of disease prognosis or certainty

### Step two: synthesis preparation

Preparing to synthesize two or more theories requires the clarification of existing theories and extracting what is useful, plausible, and relevant [26, 48]. While many theories address the concept of communication and uncertainty, the Reconceptualized Uncertainty in Illness Theory (RUIT) and the Problematic Integration Theory (PIT) are uniquely applicable to the uncertainty arising from serious LLI and the cultivation of supportive ACN-patient communication within that uncertainty. While RUIT and PIT vary in their conceptualization of uncertainty, both concur with Mishel’s definition of uncertainty as the “inability to determine the meaning of illness-related events…where the decision maker is unable to assign definite values to objects and events and-or is unable to accurately predict outcomes because suffering cues are lacking” [49–51]. However, integrating RUIT and PIT into a UCS framework requires relanguaging terms to support a caring nursing perspective.

#### Reconceptualized uncertainty in illness theory

The RUIT was adapted from Mishel’s original Uncertainty in Illness Theory (UIT). The UIT organizes uncertainty into three phases: antecedents of uncertainty, appraisal of uncertainty, and coping with uncertainty [52]. Antecedents of uncertainty include a person’s stimuli frame (discernment of a symptom pattern in one’s illness, familiarity with symptoms, and congruence of symptoms with expectations), a person’s cognitive capacity (person’s ability to comprehend or understand information), and a person’s structure providers (resource to aid and support people in interpreting their diagnosis and disease trajectory) [52].

In UIT’s appraisal section, Mishel uses the term *inference*, referring to a person’s uncertainty evaluation based on their comparisons with related situations [50]. If these comparisons, or *inferences*, are perceived as negative by a person, then uncertainty will be perceived as dangerous. Conversely, if these comparisons are optimistic, uncertainty may be viewed positively [49, 52, 53]. Mishel then utilizes the term *illusion* to address a person’s construction or reconstruction of beliefs that allow uncertainty to be evaluated as positive [50].

According to UIT, if a person perceives their uncertainty as dangerous or expected to produce a harmful outcome, then they are likely to take steps toward decreasing that uncertainty [49, 50, 53]. However, Mishel asserted that uncertainty is not always harmful or something to reduce. Uncertainty may be used as an *opportunity* or coping strategy to maintain hope [50]. Mishel clarifies that difficulties arising in patients feeling uncertain are not from the uncertainty itself but from “the ability of [their] coping strategies to manipulate the uncertainty in the desired direction…to reduce it if it is appraised to be a danger or maintain it if it is appraised to be an opportunity” [49]. Mishel further operationalized the UIT by developing Mishel’s Uncertainty in Illness Scale (MUIS). Mishel developed the MUIS to address ambiguity, inconsistency, complexity, and unpredictability in patients’ illness experiences [53]. The MUIS is a 28-item Likert-type scale ranging from five (strongly agree) to one (strongly disagree) [53]. The UIT is widely utilized in nursing research, and the MUIT provides further empirical support for many of UIT’s assertions [53–55].

After UIT’s initial publication, Mishel recognized several cultural biases inherent throughout the theory, especially its preference for certainty with an orientation toward maintaining or achieving equilibrium [49]. Therefore, Mishel addressed three primary concerns in the Reconceptualization of the Uncertainty in Illness Theory (RUIT): implicit assumptions of UIT, conflicting research findings, and blocks to advancing the UIT [49]. Subsequently, Mishel developed the RUIT using theory derivation that combined aspects of Chaos Theory with their previously developed UIT [49]. Mishel’s RUIT theorizes the experience of living with continual or chronic uncertainty [49], which aligns with those who would benefit from early palliative care.

Mishel’s incorporation of Chaos Theory into the RUIT introduces the concepts of *entropy* and *flux* into uncertainty in illness. *Entropy* refers to the degree of disorder or disorganization in a system. *Flux* refers to the continual fluctuations inherent to all systems and subsystems. By incorporating these aspects of Chaos Theory, Mishel concluded that uncertainty in one area of illness may feed back on itself and all areas of a person’s life [49]. The RUIT describes how uncertainty in serious LLIs functions as a source for nonlinear reactions that loop back on themselves, constantly generating and dissipating uncertainty [49]. Mishel’s RUIT is based on social psychology conceptualizations and heightens awareness of uncertainty as an expected phenomenon inherent to serious LLI.

Mishel also revised the appraisal-coping section of their original UIT for the RUIT. While UIT shows opportunity and danger to be parallel and indicate a linear cause-and-effect relationship based on a person’s choice, Mishel later recognized that linearity may not accurately reflect the “fluctuations that occur over the course of illness and does not consider the long-term illness situation” [49]. Therefore, Mishel (1990) expanded UIT’s theoretical statements to account for a person’s growth and self-organization as an outcome of coping with uncertainty, not solely an equilibrium-stability view.

##### Reconceptualized uncertainty in illness and unitary caring science

In addressing their previous concerns with UIT, Mishel aligns the RUIT with UCS and its unitary transformative worldview. The primary implicit assumption Mishel reconceptualizes in RUIT is a person’s adaptation to chronic illness. The UIT is a static linear theory that does not allow growth and change with uncertainty as an ever-present variable. In the RUIT, Mishel (1990) recognizes that living with continual uncertainty may result in personal growth with the experience of uncertainty shifting as it interacts with time, space, and a myriad of other variables. For example, King and Mishel (1986) found that the longer chronically ill people lived with ongoing uncertainty, the more positively they evaluated uncertainty. This constant evolution of uncertainty supports RUIT’s conceptualization that uncertainty is constantly fluctuating [49], which parallels UCS’s assertion that “healing is a dynamic process of transformation involving” seemingly small acts that have ripple effects beyond a disease process to a person’s entire world [11].

The RUIT emphasizes the person as an open system “interchanging energy with the environment” oriented toward increasing complexity instead of a rationalistic and overly simplified ideal of certainty [49], aligning with UCS’s assertion that interactions between the nurse, patient, and environment are better understood as fluctuating and interchanging energies [11]. Furthermore, conceptualizing communication as solely a back-and-forth exchange of words between nurse and patient negates other factors occurring simultaneously in a patient’s universe, from what is on the television and how the room smells to social pressure, psychological health, and financial stability.

#### Problematic integration theory

Mishel’s UIT and RUIT only tangentially consider communication. Conversely, Babrow developed the Problematic Integration Theory (PIT) because of shortcomings within the widely accepted expectancy-value theories of persuasion and action, which they felt undertheorized health communication phenomena. Babrow’s original goal was to establish communication’s significant role in a person’s problematic integration. According to Babrow, *problematic integration* is the misalignment of a person’s beliefs and expectations, where one’s expectations for what may happen inconsistently relate to what one desires, and this misalignment makes constructing meaning challenging [31, 56]. Therefore, PIT conceptualizes communication as the constructive process that forms, maintains, and transforms meaning [31].

According to Babrow (1992), people continually integrate *probabilistic* and *evaluative* orientations and link them to “broader complexes of knowledge, feelings, and behavioral intentions” [57]. When probabilistic and evaluative integration is uncomplicated with clear probable outcomes based on a person’s evaluation of the situation, problematic integration is not likely to develop [57]. For example, a person diagnosed with stage one breast cancer who trusts the guidance of their doctors has one clear course of treatment and a high likelihood of a favorable outcome will have minimal uncertainty with integrating the information and deciding on a treatment course. This person has converging expectations and evaluations of the probability of a positive outcome. Therefore, their probable outcomes are easily integrated with their relevant beliefs, attitudes, and intentions. However, “integration becomes more difficult as probability and evaluation diverge, probability becomes less clear, or ambivalence arises” [57].

Babrow depicts uncertainty as one of four forms of *problematic integration*: uncertainty, divergence, ambivalence, and impossibility/certainty [58]. The PIT states that uncertainty exists when determining the probability of something positive or negative occurring is difficult [57]. However, *Divergence* occurs when there is a discrepancy between what a person wants (evaluative) and what is likely to occur (probabilistic) [51, 56, 57, 59]. For example, a patient with a progressive LLI may want to be discharged from the hospital better than when admitted (evaluative) because they hope the hospital can make them better. However, the likelihood is that with each hospitalization, the patient will be discharged slightly more deteriorated (probabilistic). *Ambiguity* occurs when the probability of an event or issue is unknown or uncertain [51, 56, 57, 59]. For example, the disease trajectory for many chronic diseases is uncertain. While a patient diagnosed with heart failure may insist on knowing exactly what they can expect over the next five years, the nurses and doctors cannot guarantee definite outcomes. *Ambivalence* is created when a person must choose between two substantively different but mutually exclusive choices, with the outcome of each holding a similar value. Ambivalence can also occur when a person has contradictory feelings about a single person, place, or thing [51, 56, 57, 59]. For example, a cancer patient may have several treatment options, all with potentially positive outcomes and adverse side effects, and therefore feel uncertain regarding the best course of action. *Impossibility* occurs when a person feels an undesirable outcome is inevitable. This impossibility can manifest as feeling there is zero probability that a positive event will happen or feeling that there is a 100% probability that something negative will occur. Like Mishel, Babrow clarifies that people may seek uncertainty when facing impossibility because uncertainty allows them to maintain hope [50, 56].

Through these concepts of problematic integration, PIT attempts to explain how communicative constructions are significant sources for coping with uncertainty. According to the PIT, communicating with people regarding their uncertainty requires knowledge of their worldview (epistemological or ontological). Those with epistemologically leaning worldviews are more likely to see the world as rooted in human understanding and the inherent limitations to that knowledge [31, 56]. Therefore, a person with an epistemological worldview is more likely to seek information and education to reduce uncertainty [31, 56]. For example, a patient with multiple sclerosis feeling uncertain about their future health and ability to stay physically independent may continually request further testing to gain more knowledge about their disease process while researching and requesting information on treatment options. More information may give this person more comfort. Contrarily, a person with a more ontologically leaning worldview is more likely to see the world as something that is never perfectly knowable. Those with stronger ontological perspectives tend to emphasize the complexities of causality in diverse relationships and the unpredictability of random influences on these associations [31, 56]. For example, the same person diagnosed with multiple sclerosis but with an ontological worldview may focus on life’s unpredictability to reduce uncertainty. This person may have the mindset that they could get hit by a car tomorrow. As long as they take the medication the doctor prescribes, they will try to focus on their friends and family as positive distractions from a world filled with uncertainty for everyone. The time nurses spend with their patients allows them to better understand their patients’ worldviews and can support their communication choices regarding palliative care.

While Babrow (1992) agrees with Mishel’s definition of uncertainty in UIT, they offer a social constructionist viewpoint of uncertainty in contrast to the psychological perspective of UIT [31]. However, when Mishel reconceptualized their worldview in RUIT to one of probabilistic thinking, they moved closer to Babrow’s social constructionist worldview.

##### Problematic integration theory and unitary caring science

The uniqueness of the nurse’s perspective makes nursing a valuable contributor to PIT. While Babrow acknowledges the importance of nursing for utilizing the PIT in practice, they equate this importance mainly to the amount of time nurses spend with patients and not their unique worldview [51]. Therefore, PIT research within the discipline of nursing must adjust to incorporate nursing’s caring language and perspective. The PIT illuminates the essential and ever-present communication process inherent to serious LLIs [57, 58]. By illustrating this communicatory process, PIT enhances the nurse’s ability to understand their patients’ uncertainty and compassionately think through alternative approaches to patient care and communication. A better sense of their patients’ uncertainty and vulnerability fosters increased empathy and compassion, facilitating UCS’s concept of authentic presence [11]. Recognizing that people are constantly forming and reforming their value structures emphasizes the nurse’s need to continually reintegrate their patients’ changing perspectives and accommodate their communication in response.

Babrow’s use of the word *problematic* to describe natural and expected feelings associated with a severe LLI pathologizes a normal process of wellbecoming. Therefore, the vulnerability generated by what Babrow termed *problematic integration* must be relanguaged through a UCS lens to support patients in the vulnerability of uncertainty. Babrow’s assertion that “*meaning is problematic* [italic added for emphasis] when it is difficult to synthesize or integrate values or desires with beliefs or expectations” can be reworded to *meaning creates vulnerability* “when it is difficult to synthesize or integrate values or desires with beliefs or expectations” [51]. PIT languages the uncertainty arising from difficulty integrating values or desires with beliefs and expectations as a problem nurses can solve through communication. Conversely, a UCS perspective emphasizes that vulnerability is inherent in uncertainty and is a natural emotion. Lombard and Horton-Deusch further emphasized the UCS view that “respect for human vulnerability preserves dignity and that respect was an important part of helping-trusting relationships and creating a healing environment” [60]. While a nurse still may work to reduce their patients’ feelings of vulnerability, those feelings are not deemed harmful, wrong, or shameful because the experience of vulnerability is often a necessary step for finding meaning, value, support, and a way forward in their serious LLI journey [13].

### Step three: synthesis

The synthesis process overlapped with synthesis preparation and involved breaking down PIT and RUIT into their simplest propositions. Then, comparing those propositions for convergence, divergence, and meaningful cohesion [26]. By condensing Babrow’s types of *problematic integration* within uncertainty, we could remove the term *problematic integration* entirely, thereby removing unnecessarily negative connotations to a naturally adaptive process of probabilistic thinking in uncertainty. The MUIS provided additional insight into variables of uncertainty through its measurement of inconsistency, complexity, and unpredictability [53]. Integrating the converging propositions in PIT, RUIT, and UCS created six distinct variables within our communication conceptual framework (divergence, ambivalence, impossibility, complexity, inconsistency, and unpredictability).

- **Divergence** refers to discrepancies between what a person wants and what is likely to occur. However, what a person wants is much more complex than curative interventions. Therefore, divergence may include existential concerns, such as finding meaning and maintaining control.
- **Ambivalence** is created when choosing between two different, mutually exclusive choices, with the outcome of each holding a similar value. Ambivalence is a complex variable of emotional uncertainty that may leave people conflicted or unsure of not just the “correct” clinical decision but also conflicted in their beliefs and values.
- **Impossibility** addresses the feeling that an undesirable outcome is inevitable. However, how a person defines an “undesirable outcome” is individualized and influenced by their worldview and existential beliefs.
- **Complexity** refers to the multiple variables and options to consider throughout a chronic illness that prevent a simple decision-making process. Complexity also refers to the numerous and variable emotions accompanying LLI, leading to emotional uncertainty that may make answering the question of “How are you?” an unexpectedly overwhelming query.
- **Inconsistency** is when a person’s expectations are inconsistent with what is subsequently experienced. Inconsistency may occur with an unexpected physical outcome. However, inconsistency may also arise with fluctuating emotions and unanticipated emotional responses.
- **Unpredictability** is a variable of uncertainty often occurring when the probability of an event or outcome is unknown. Unpredictability also encompasses a person’s emotional uncertainty throughout their LLI and includes the surfacing of unexpected emotions or expected emotions at unpredictable times.

### Step four: synthesis refinement

**Fig. 3:**
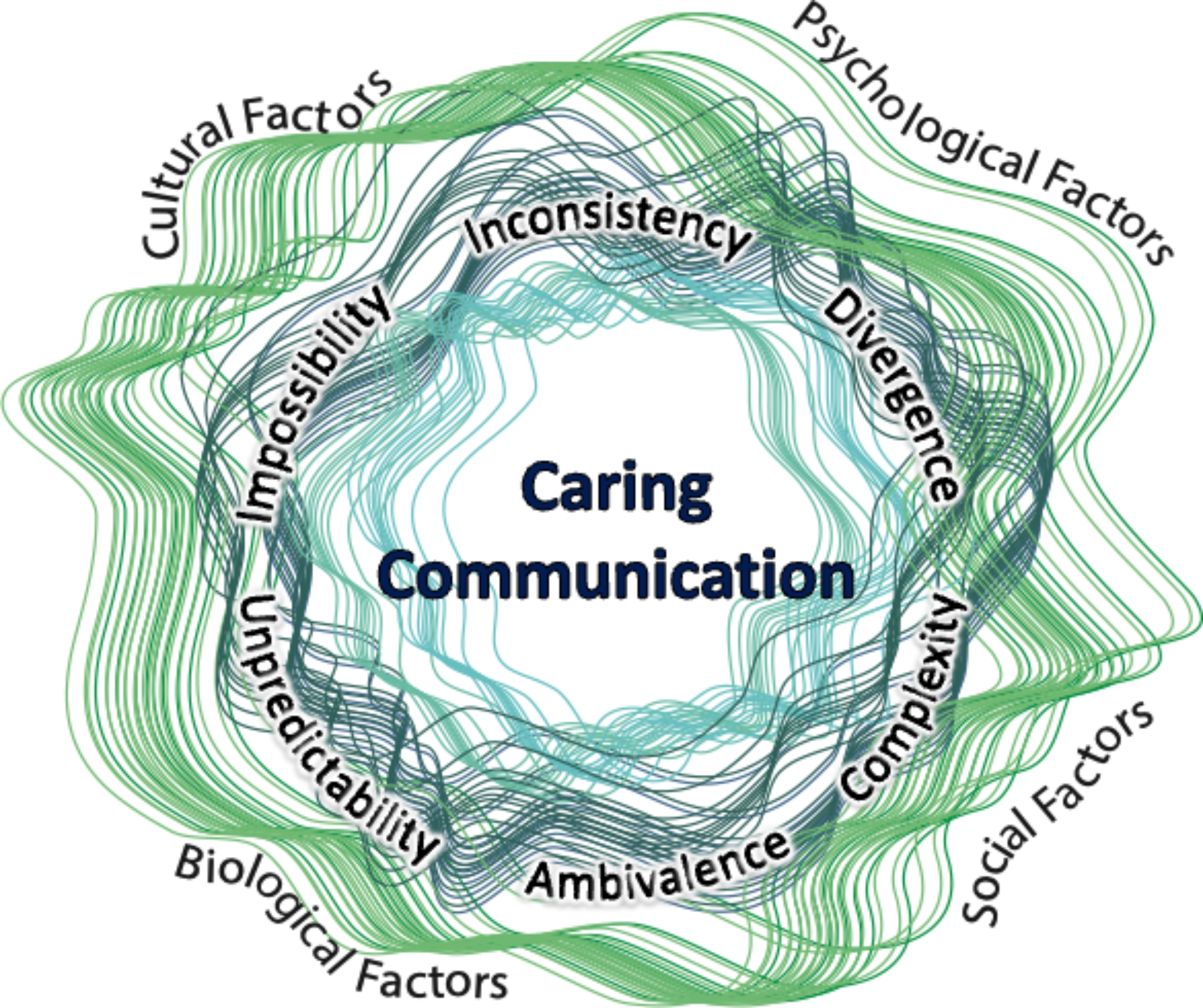
Nursing communication framework of uncertainty in life-limiting illness

Synthesis refinement is the final step in Turner’s theory synthesis and involves examining previously identified convergent processes to reconceptualize the original theory propositions into a new and overarching framework [26]. Purposefully, our refined conceptual framework has no start or end point. Communication and uncertainty are ever-present, dynamic, and in constant flux. Therefore, the trajectory of uncertainty in serious LLI is represented by multiple fluctuating lines that often overlap, along with the types of uncertainty and factors of uncertainty (Fig. 3).

The factors shown in this framework are drawn from an ecological approach to palliative care, which utilizes the biopsychosocial model to address the many aspects of one’s life and social determinants of health [34]. Within this theory synthesis, PIT and RUIT converge in their conceptualization of uncertainty as something complex, individualized, impactful beyond disease prognosis, and inclusive of emotional and existential wellbeing [61–64]. These factors are represented along the outermost light green fluctuating circle. Cultural factors include Babrow’s differentiation between a person’s ontological versus epistemological worldview. Questions arising within one’s worldview contribute to existential uncertainty as patients continually face questions of life, death, identity, meaning, and choice [56, 57, 64]. Culture also includes a person’s religious or spiritual beliefs and the social behaviors considered acceptable or taboo in their communities [49, 65]. Social factors reflect a person’s readily available choices based on their community, income, and education. A few social factors influencing uncertainty are access to and belief in a credible authority, social support, health literacy, and financial stability [49, 50, 52, 53]. Psychological factors of uncertainty are the elements of a patient’s personality and psychological state that influence how a person thinks. In uncertainty, these psychological factors can include a sense of mastery or empowerment, a person’s locus of control, and depressive symptoms such as learned helplessness [49, 52, 53, 66, 67].

The different variables of uncertainty are represented along the dark green middle section of the framework. Again, uncertainty is never static, and a person with a serious LLI will likely experience several types of uncertainty at different intensity levels throughout their disease trajectory. Knowing the variables of uncertainty can help nurses understand their patient’s worldviews and feelings of vulnerability. Nurses who better understand their patients’ concerns can subsequently adapt their communication along with their patient’s needs [22, 51]. However, none of these factors occur in isolation from one another. Biological factors such as symptom familiarity overlap with the social factor of health literacy, which may, in turn, influence a person’s worldview, existential beliefs, and emotional state. Factors influencing uncertainty are constantly in flux, interacting with each other and the environment [49].

Caring communication is central within this framework, represented by the light blue/green fluctuating lines. The fluctuation of the lines represents the continually changing and adapting communication process. The decision to keep all the lines of this framework in the same color palate was intentional and meant to represent the all-encompassing nature of communication for patients experiencing uncertainty in serious LLIs. No one communication process is listed in this model because the type of communication needed for every variation of the patient experience with uncertainty is innumerable. Instead, communication is an inseparable concept integrated with every factor and variable of uncertainty.

The theoretical framework resulting from our theory synthesis is not a linear appraisal of communication during uncertainty in illness. Instead, this framework represents the integrality of many facets of uncertainty for a person with a serious LLI (the person, the variables of uncertainty, and the communicatory process). The converging propositions of RUIT and PIT conceptualize uncertainty communication in a way that clarifies it as more than a simple cause-and-effect process but a series of events happening simultaneously. Therefore, ACNs’ can never presuppose a singular solution to a person’s uncertainty. Instead, ACNs’ can learn about the integrated and variable nature of uncertainty, their patients, and communication techniques, allowing them to be a positive communicatory factor within the flux of uncertainty.

## Discussion

ACN-patient communication addressing serious LLI and palliative care likely includes ongoing conversations about an uncertain future. Therefore, this paper aimed to (a) provide a focused literature review on nurse palliative care communication addressing chronic uncertainty in LLI, (b) define the RUIT and PIT within a nursing UCS philosophical worldview, and (c) synthesize these theories and literature review into a unique theoretical framework for early palliative care communication in nursing.

### Implications for nursing practice

This theory synthesis is vital for nursing practice because it brings a UCS philosophy and nursing perspective to uncertainty in LLI. This nursing perspective alters the communication goals surrounding palliative care from solely a physician-driven model of goals of care and prognosis to one of supporting and caring for patients within their existing uncertainty and vulnerability [68, 69]. Drawing from UCS’s philosophical origins, our resulting theoretical framework provides practicing nurses with another “way of knowing” their patients by incorporating uncertainty as an expected variable requiring the nurse’s recognition and understanding [11].

Research surrounding barriers to palliative care communication in nursing practice demonstrates a widespread perception that the patients and families are not ready or willing to have palliative care discussions [70, 71]. However, further research on patients’ and families’ satisfaction with palliative care communication demonstrates high satisfaction levels when palliative care discussions occur [21]. Furthermore, MUIS studies show decreased uncertainty in patients who felt they received clear, supportive communication, regardless of their disease prognosis [55, 72]. These findings contradict nurses’ perceptions that patients are not open to discussing the uncertainty surrounding their serious LLI. This misconception drives the need for further nursing research and education regarding how ACNs can confidently initiate caring palliative and uncertainty discussions in practice.

### Implications for nursing education

Communicating about an uncertain future instead of presenting a concrete solution is not easy, prescriptive, or straightforward. Therefore, ACNs need specialized communication education that prepares and empowers them to discuss the omnipresent uncertainty in everyone’s lives regardless of illness severity or prognosis. Subsequently, this theory synthesis can also be viewed as an educational tool allowing nurses to gain greater insight into their patients’ clinical, emotional, and existential uncertainty. With this increased knowledge, nurses can better select appropriate caring communication strategies for patient-specific situations.

Health communication relies heavily on frameworks to translate theories to practice, and evidence-based communication guides can be an important resource for nurses navigating their patient’s chronic uncertainty. For example, the SPIKES framework for breaking bad news to patients [73], the BATHE framework to screen for situational anxiety and depression [74], the NURSE framework for responding to emotional cues [75], and the SURETY framework for nonverbal communication [76] provide nurses reassuring guidance for various communication challenges. However, frameworks are often context-specific, and those geared toward physician-patient interaction or specific patient populations may not be appropriate for all ACN-patient interactions. Furthermore, many frameworks ascribe to the knowledge deficit model of health education and uncertainty reduction, where healthcare providers and educators address uncertainty by further educating patients on their disease or prognosis [68, 77]. Evidence-based research does not support most knowledge deficit models because of their ineffectiveness in overcoming people’s preexisting biases that formed their initial health and wellness beliefs [68, 78]. According to Wang et al. [78], the biases that filter how we process information also allow us to package new information in ways that reaffirm our existing beliefs [78]. Therefore, this theory synthesis and resulting framework provide nurses additional knowledge and support when determining the *context* of uncertainty for their patient interactions without prescriptively attempting to change the patient’s belief systems.

### Implications for nursing research

None of the studies meeting our inclusion criteria contained longitudinal data on how uncertainty evolved in people with chronic LLI over months or years. Therefore, longitudinal research focusing on how uncertainty develops and transforms over time in patients with serious LLI is a promising avenue for future nursing research. Ideally, this longitudinal research would start when a person is first diagnosed with an LLI and continue at regular intervals for their entire disease trajectory.

Furthermore, future nursing research must concentrate on interventions that take advantage of nurses’ unique roles and relationships in patient care. Nurses rarely participate in formal goals of care meetings and, even more rarely, call for them themselves [79, 80]. Yet, nurses address patients’ goals of care almost every time they enter the room, from the more explicit act of writing a goal of the day on a patient’s whiteboard to less explicit goals, such as assisting a patient in walking to the bathroom instead of using a bedpan. Formal conversations surrounding end-of-life care rarely occur in bedside nursing practice, not because those conversations are outside ACNs’ scope of practice but because the opportunity for that conversation may not arise during a busy nursing shift [80, 81]. Therefore, a promising avenue for future nursing research includes narrative methods in nursing palliative care communication [82]. Nurses often build close patient relationships through ongoing care, a prerequisite for effective narrative interventions [82]. Therefore, integrating narrative palliative care communication techniques into ACNs’ conversational toolbox may provide an informal yet effective avenue for introducing the concept of palliative care.

### Limitations

This theory synthesis has a few limitations that should be acknowledged. First, the limited number of reviewers for the initial PRISMA literature search increases the risk of bias. Furthermore, the authors’ reliance on English-language sources for this synthesis likely restricted the breadth of perspectives and information available for analysis. Future studies will benefit from a broader review team to enhance robustness and reliability. Second, the scope of the PRISMA review was constrained by the limited amount of existing literature on ACN early palliative care communication, which highlights the need for further investigation into this specialized area of nursing communication. Third, no review protocol was registered for this study’s PRISMA literature review. Lastly, there was a notable gap in research concerning how nurses communicate about uncertainty and the context or environment in which nurses communicate. This gap limited the PRISMA literature review results and emphasized the need for further research on how ACNs contribute to early palliative care discussions for patients with LLIs.

## Conclusion

This paper integrates three theoretical frameworks to describe the phenomenon of patient uncertainty in serious LLIs and the role of ACN communication within that uncertainty. By advancing our understanding of ACN’s unique patient care role and humanizing communication during chronic uncertainty, we can provide ACNs with improved education, equipping them to confidently have early palliative care discussions. Future implementation nursing research focusing on enabling nurse-initiated early palliative care communication will support patients’ values and dignity throughout more than a single hospital stay but their entire life and disease trajectory.

## Electronic supplementary material

Below is the link to the electronic supplementary material.

Supplementary Material 1

## Data Availability

No datasets were generated or analysed during the current study.
